# An Updated Review on the Psychoactive, Toxic and Anticancer Properties of Kava

**DOI:** 10.3390/jcm11144039

**Published:** 2022-07-12

**Authors:** Rita B. Soares, Ricardo Jorge Dinis-Oliveira, Nuno G. Oliveira

**Affiliations:** 1 Research Institute for Medicines (iMed.ULisboa), Faculty of Pharmacy, Universidade de Lisboa, 1649-003 Lisbon, Portugal; soares.rita@edu.ulisboa.pt; 2 TOXRUN—Toxicology Research Unit, University Institute of Health Sciences (IUCS), CESPU, CRL, 4585-116 Gandra, Portugal; 3 Department of Public Health and Forensic Sciences, and Medical Education, Faculty of Medicine, University of Porto, 4200-319 Porto, Portugal; 4 UCIBIO-REQUIMTE-Applied Molecular Biosciences Unit, Laboratory of Toxicology, Department of Biological Sciences, Faculty of Pharmacy, University of Porto, 4050-313 Porto, Portugal; 5 MTG Research and Development Lab, 4200-604 Porto, Portugal

**Keywords:** kava, cancer, toxicity, toxicokinetics, interactions, clinical uses, anxiety

## Abstract

Kava (*Piper methysticum*) has been widely consumed for many years in the South Pacific Islands and displays psychoactive properties, especially soothing and calming effects. This plant has been used in Western countries as a natural anxiolytic in recent decades. Kava has also been used to treat symptoms associated with depression, menopause, insomnia, and convulsions, among others. Along with its putative beneficial health effects, kava has been associated with liver injury and other toxic effects, including skin toxicity in heavy consumers, possibly related to its metabolic profile or interference in the metabolism of other xenobiotics. Kava extracts and kavalactones generally displayed negative results in genetic toxicology assays although there is sufficient evidence for carcinogenicity in experimental animals, most likely through a non-genotoxic mode of action. Nevertheless, the chemotherapeutic/chemopreventive potential of kava against cancer has also been suggested. Both in vitro and in vivo studies have evaluated the effects of flavokavains, kavalactones and/or kava extracts in different cancer models, showing the induction of apoptosis, cell cycle arrest and other antiproliferative effects in several types of cancer, including breast, prostate, bladder, and lung. Overall, in this scoping review, several aspects of kava efficacy and safety are discussed and some pertinent issues related to kava consumption are identified.

## 1. Introduction

Kava is a shrub plant with psychoactive properties commonly used in the South Pacific islands that has recently awakened the interest of the scientific community for its potential in cancer treatment. Kava is obtained from the pulverized dried roots of different species of *Piper methysticum* Forst and can be translated as “intoxicating pepper” [1]. The term kava means “bitter” [2] in the local language and is usually used when referring to the beverage obtained from this plant. However, since the plant began gaining popularity in the Western regions, the name kava has also been associated with pills or herbal medicines [3]. As described by Humberston et al. [4], there are several synonyms of kava, depending on the region of the globe. In fact, there are numerous traditional or common names such as kava kava, ava, awa, intoxicating pepper, kava pepper, kava root, kawa, kew, sakau, tonga, and yagona. Additional denominations for kava can be found at the National Toxicology Program (NTP) [5] and at the International Agency for Research on Cancer (IARC) reports [6].

The beverage kava had a strong ceremonial connotation, although it is now mainly used as a social beverage. Nevertheless, there are some countries and regions in the Pacific, namely Tonga, Fiji, and Pohnpei, where kava ceremonies are practiced as a vital part of local culture [7]. Why kava spread throughout the Pacific islands can be explained by the fact that this plant displays psychoactive properties, bringing a sensation of calm and relaxation, thus becoming an appealing beverage to both locals and tourists. It is believed that kavalactones, the main constituents of kava, are responsible for the relaxation properties [8]. Given these characteristics, kava has reached Western countries as an option to cope with anxiety disorders and stress as well as being used as a pain reliever [7]. Importantly, in recent years, kava has also been studied as a potential bioactive agent in the cancer field [1,2,9].

The usage of herbal and natural medicines is quite often erroneously associated with the absence of adverse side effects. In the context of kava ingestion, there are several reports indicating its potential for the development of hepatotoxicity [8], skin rash [10], gastrointestinal problems and headaches [11], among other adverse effects that we address throughout this review. Nevertheless, the real impact of kava in terms of safety needs further elucidation. There are several factors involved in this complex issue that can influence the putative beneficial effects of this millenary mixture. In fact, some aspects such as the quality of the plant, mode of extraction, solvents used, and the chemical composition presented are crucial points to consider.

This review aims at integrating safety and efficacy issues of kava, providing some historical background, and reviewing a number of chemical aspects involved. Additionally, the absorption, distribution, metabolism and excretion (ADME) of kava constituents as well as their pharmacodynamics and potential clinical beneficial effects, particularly in the scope of cancer, will be further reviewed and updated.

## 2. Materials and Methods

A comprehensive literature search of studies in English was conducted between January 2021 and May 2022 to identify articles (i.e., original and reviews) to be included in the present work. The search was carried out in PubMed (U.S. National Library of Medicine) without a limiting period, although particularly focusing on reports published in the last decade. The keywords used were “kava”, “toxic”, “genotoxic”, “toxicokinetics/pharmacokinetics” “hepatotoxic”, “psychotropic” and “cancer”. Specific kava constituents were also searched. In addition, the abovementioned articles, as well as textbooks and technical reports on kava, were further reviewed to identify additional relevant publications.

## 3. Historical and Social Perspectives on the Consumption of Kava

The kava plant is native to Vanuatu, domesticated for the first time approximately 3000 years ago and brought to Oceania by Austronesian colonists [3]. It is often said that Captain James Cook introduced kava to Europe in the 1770s. Nevertheless, as reviewed by Norton and Ruze [7], this plant had already arrived in Europe approximately 150 years earlier by Dutch explorers. In the scientific field, George Foster provided a detailed description of kava, becoming the first scientist to register the effects of kava on the skin [7].

The consumption of kava consisted of mixing kava root in different ways (i.e., crushed, grated or chewed), depending on the region of the Pacific where the ritual was performed, and mixing it with water or coconut milk. The greyish and turbid beverage obtained might discouraged foreign people to drink it [7]. Traditionally, kava ceremonies were typically held in major events such as weddings, funerals, births, and on the arrival of royalty, with this mixture serving as an honor [10]. Independently of being a formal or informal ceremony, these gatherings were exclusively made of a collective of males, and could last from 12 h, e.g., an informal gathering [12], to up to 4 days, in the case of a funeral [10]. Importantly, nowadays, most Pacific islanders drink kava not only for ceremonial and medicinal purposes, but also to help them socialize, reaffirm relationships and status and as an alternative to ethanol [12].

Despite its current usage in different disorders and diseases, kava became popular in the 1990s, as an attractive recreational drug, reaching peak popularity in 1998 [3]. This led to an increase in reports mentioning kava as the main cause of different types of liver damage, thereafter carefully considered for its hepatotoxicity. In view of this, the German organization Federal Institute for Drugs and Medical Devices (BfArM) decided to ban all kava products in 2002, and many countries followed the same decision due to safety concerns [3,4]. In addition, the U.S. Food and Drug Administration (FDA) decided to investigate if these products were indeed responsible for public health problems, and after reviewing the available cases, decided to issue a statement on the products containing kava, albeit not banning them [3]. Later, in 2014, two German administrative courts concluded that the relationship between kava ingestion and liver damage was not well established in most cases [13].

Today, in the Pacific islands, kava still has traditional importance, used in ceremonies and rituals. However, there are some differences compared to its former use. Previously, as aforementioned, kava ceremonies were only performed and attended by men, while now women are also progressively drinking kava in informal rituals [1]. Moreover, in the past, kava was consumed in large events rather than in small ceremonies, while now the opposite is more common, since men start drinking kava at younger ages [12] and it is used for socializing purposes.

## 4. Chemical Aspects of Kava

### 4.1. Chemical Composition of Kava

**Figure 1:**
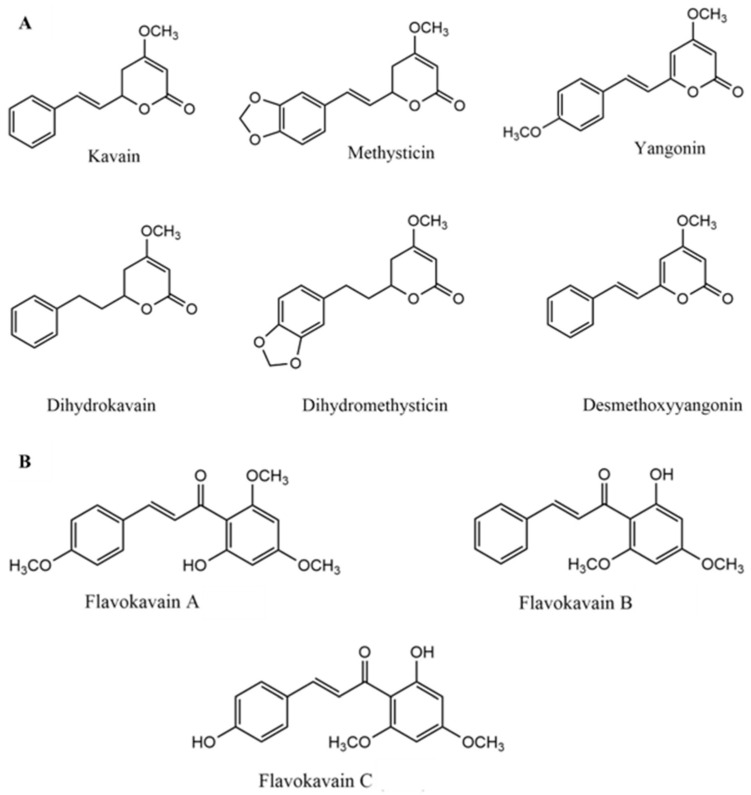
Chemical structures of the main kavalactones (**A**) and flavokavains (**B**).

The chemical composition of kava can be affected by several factors. The most important include the type of cultivars, which can present different chemicals, the parts of the plant used, and solvents for extraction. The main constituents responsible for the biological activity of kava are lipophilic lactones called kavalactones (or kavapyrones) that possess a α-pyrone skeleton with aromatic stiryl or phenylethyl substituents at the 6th position [14]. Nineteen different kavalactones have been identified [15] which can be metabolized in the liver by cytochrome P450 enzymes (CYP450) [16]. However, only six kavalactones are considered responsible for approximately 96% of the pharmacological activity: kavain, dihydrokavain, methysticin, dihydromethysticin, yangonin and desmethoxyyangonin (Figure 1A) [2,17,18]. Methysticin is thought to help in the neuroprotection against ischemia and, in combination with dihydromethysticin, may reduce brain infarction in mice [19]. Moreover, kavain, dihydrokavain and methysticin are considered to be the most important kavalactones for the effects observed in the central nervous system (CNS) [20]. Yangonin has been suggested to display hepatoprotective effects against cholestatic liver injury [21].

Minor constituents consist of chalcones (flavokavains A, B and C) (Figure 1B), amino acids and minerals such as aluminum, iron, magnesium, calcium, and sodium [20]. Although flavokavains A, B and C represent a minimum part of kava chemical composition, they have been described as displaying important biological roles. Flavokavain B induces apoptosis in cancer cells by generating reactive oxygen species (ROS) [22] and might be the main constituent responsible for hepatotoxicity [23]. In turn, flavokavain A might exhibit anticancer properties [24].

The part of the plant used is important for the chemical composition. Kava extracts can be prepared using different parts of the plant, i.e., roots and rhizomes, stems and leaves, which results in different outcomes and side effects. The content of kavalactones and flavokavains is higher in the roots, being almost absent in the aerial parts of the plant, which are in turn rich in toxic alkaloids, such as pipermethystine [16]. The roots and rhizomes are the only parts to have been tested in clinical trials and peeled roots may reduce the likelihood of hepatotoxicity. The stems have a high alkaloid content, which may lead to cytotoxicity [2]. Nonetheless, the peelings of the basal stems are used for pharmaceutical purposes [25]. Finally, the leaves are not involved in kava preparations, but can be used in tea preparations [26]. Dinh et al. [27] reported that extracts from kava leaves interact strongly with CNS receptors in vitro.

The chemical composition of kava, especially the concentration of kavalactones and flavokavains, and consequent biological properties, are directly related to the cultivar. In 2002, the Kava act nr.7 was issued, which classified the existing cultivars and fit them into four categories: noble (*n* = 28), two day (also known as “tudei”, *n* = 128), medicinal (*n* = 79) and wichmannii (*n* = 12) [28]. Kava from noble cultivars could be exported since this is the only one capable of providing the desired effects in a safe and predictable way [29]. This type of cultivar has higher concentrations of kavain, therefore offering a higher quality and better anxiolytic properties when compared with other cultivars [2].

The two-day cultivar presents high levels of dihydromethysticin and dihydrokavain, which causes a “hangover” effect, characterized by the presence of nausea and headaches [30]. This cultivar is denominated “two-day” because the psychoactive effects can last up to two days, which makes it attractive for recreational use. It has also high levels of flavokavain B, leading to a higher probability of hepatotoxicity [28]. Medicinal and wichmannii cultivars are less studied. Medicinal cultivar has a history of beneficial properties among Pacific herbalists. However, it has only been used in medicinal and dietary products, which might explain the relationship between liver toxicity and patients that used these extracts [25]. Wichmannii cultivar causes psychological effects that are considered too strong among the native habitants of Pacific Islands. It has elevated levels of all three flavokavains, yangonin and desmethoxyyangonin, similarly to the two-day cultivar [28].

Finally, the solvents used for extraction and the methods used to characterize kava extracts are of utmost importance to obtain a detailed fingerprint of each extract used and is needed to conduct future investigations.

### 4.2. Overview of the Methodologies Used for the Chemical Analysis of Kava

The World Health Organization (WHO) published a monograph recommending the use of high-performance liquid chromatography (HPLC)—electrospray mass spectrometry to obtain qualitative analytical profiles and HPLC as the standard method for quantitative analysis [31]. Reverse-phase HPLC is considered advantageous, since the results obtained are highly reproducible, especially using 2-propanol as eluent, and because it displays a very low detection limit and does not require an internal standard [14]. More recently, other authors reported the use of thin-layer chromatography (TLC), liquid chromatography–mass spectrometry (LC–MS), and UV–vis spectrometry [2]. In addition to these analytical methods, other techniques arose in a way to provide possible solutions to the standardization problem. One of these techniques is the qualitative and quantitative analysis of multicomponents by single-marker methods (QAMS). This method is based on the relationship between the quantity of components and the detector response, calculating the relative correction factor (RCF). This quantity (mass or concentration) will be proportional to the detector response, which includes peak area, height, and absorbance. One of the advantages of this method is that the multicomponents can be determined using only one reference [32]. Ultra-performance liquid chromatography high-resolution tandem mass spectrometry (UPLC–MS/MS) has also shown high selectivity and specificity, which can be useful to characterize kava products and to obtain more information on its pharmacokinetics data in animals and humans [33]. Finally, the usage of high-performance liquid chromatography interfaced to a diode array detector (HPLC–DAD) allowed the quantification of total kavalactone content and its distribution profile [34].

## 5. Toxicokinetics and Drug Interactions

### 5.1. ADME Aspects of Kava

Currently, kava has two different modes of oral consumption. The semi-traditional method of ingesting kava as a beverage is still used. In this way, kava is extracted using mostly water and served in a coconut shell, resembling the traditional way in the Pacific Islands, which involves high doses and a lack of control of the dosages used. This beverage can only be found in bars dedicated to this drink, known as kava bars. The second method of consumption is focused on the nutraceutical and medicinal properties of kava, with tablets being the most common pharmaceutical form. It is used with the specific aim to treat a certain disease, particularly anxiety disorders [1].

Since few studies have been published regarding toxicokinetics, a full compilation of data is provided in this review, also exploring the potential of interaction with other co-administered compounds to clarify the risk of kava-induced liver injury [35,36,37,38]. The results from disposition studies on radiolabeled kavain in male F344 rats revealed that it is rapidly absorbed and distributed to tissues following oral administration [5]. Urinary and fecal excretion accounted for 77% and 14% of the administered dose, respectively, and the total radioactivity in tissues was less than 0.4% at 72 h [5]. Keledjian et al. [39] employed GC–MS spectral analysis to determine the uptake of dihydrokavain, kavain, desmethoxyyangonin, and yangonin into the male Balb/c mouse brain at 5, 15, 30, and 45 min following intraperitoneal administration. After 5 min, dihydrokavain and kavain attained maximum concentrations of 64.7 and 29.3 ng/mg wet brain tissue, respectively, and were then eliminated rapidly. By comparison, both desmethoxyyangonin and yangonin reached lower maximum concentrations, but were eliminated slowly from brain tissue [39]. The rapid brain penetration of the lactones observed were consistent with the high lipid solubility of these compounds. Rasmussen et al. [40] also demonstrated that the kava pyrones in general have high lipid solubility. This chemical profile is also evident in the production of the concentrates. Indeed, when kava is dissolved in an ethanol/water combination, the resultant extract contains nearly 30% kavalactones, whereas alternatively when kava is extracted via an acetone–water mix, there is a mean kavalactone concentration of approximately 70% [41]. Moreover, differences were also registered in the administration of crude resin in comparison to individual compounds, suggesting synergistic pharmacokinetic interactions between constituents. Specifically, when the uptake of dihydrokavain, kavain, desmethoxyyangonin, and yangonin was measured in mouse brain as components of kava resin, the absorption was greater than when they were given as isolated materials [39].

The metabolism of the three 5,6-dihydro-α-pyrones (i.e., dihydrokavain (7,8-dihydrokavain), kavain, and methysticin) and the two α-pyrones (i.e., 7,8-dihydroyangonin and yangonin) in male albino rats was studied by Rasmussen et al. [40]. Regarding dihydrokavain, in addition to the parent compound, nine metabolites were identified, six of them being hydroxylated (three mono- and three di-hydroxylated) metabolites and three being ring-opened metabolites, in a nearly 2:1 ratio. The 8-hydroxydihydrokavain, 12-hydroxydihydrokavain (the most abundant metabolite), 11,12-dihydroxydihydrokavain, 4-hydroxy-6-phenylhexan-2-one, 4-hydroxy-6-hydroxyphenyl-hexan-2-one, and hippuric acid had their structure fully identified. No metabolites were identified in feces or bile. Nevertheless, a small amount of unchanged 7,8-dihydrokawain was present in the feces [40]. Compared to dihydrokavain, lower amounts of urinary metabolites kavain were excreted following its administration but both hydroxylated and ring-opened products were generated. Eight metabolites were identified: *p*-hydroxybenzoic acid; 4-hydroxy-6-phenyl-5-hexen-2-one, hippuric acid, 4-hydroxy-6-hydroxyphenyl-5-hexen-2-one, *p*-hydroxykavain, *p*-hydroxydihydrokavain, hydroxykavain, and *p*-hydroxy-5,6-dehydrokavain. In addition, there were two unidentified metabolites. Large amounts of unchanged kavain were identified in the feces. The metabolism of methysticin produced only two urinary metabolites, *m*,*p*-dihydroxykavain and *m*,*p*-dihydroxydihydrokavain, both in small amounts. Apparently, these metabolites were formed by demethylenation of the methylenedioxyphenyl moiety. Unchanged methysticin was also identified in feces [40]. Regarding the urinary metabolites of the α-pyrones, 7,8-dihydroxyangonin and yangonin, all reported metabolites were formed via *O*-demethylation and no ring-opening products were detected. Specifically, three urinary metabolites, *p*-hydroxy-5,6-dehydro-7,8-dihydrokavain and two dihydroxy-5,6-dehydro-7,8-dihydrokavains, were identified from the metabolism of 7,8-dihydroxyyangonin. The *p*-hydroxy-5,6-dehydro-7,8-dihydrokawain was the major urinary metabolite. Regarding yangonin, three urinary metabolites were found, with the structures of dihydroxy-5,6-dehydro-7,8-dihydrokavain and *p*-hydroxy-5,6-dehydrokavain (the predominant) being fully clarified [40].

Nine kavalactones (i.e., dihydrokavain, kavain, desmethoxyyangonin, tetrahydroyangonin, dihydromethysticin, 11-methoxytetrahydroyangonin, yangonin, methysticin, and dihydromethysticin) were identified in urine following kava ingestion [42]. The authors suggested that demethylation of the 12-methoxy substituent in yangonin, hydroxylation at C-12 of desmethoxyyangonin aromatic ring and reduction of the 3,4-double bond and/or demethylation of the 4-methoxyl group of the alpha-pyrone ring system, represent the major metabolic routes of kava. As compared to the metabolism patterns occurred in rat reported by Rasmussen et al. [40], no dihydroxylated metabolites of the kava lactones or products from ring opening of the alpha-pyrone ring system were identified in human urine. Enzymatic demethylation of 7,8-dihydromethysticin followed by ring opening of the alpha-pyrone ring, and rearrangement was suggested to contribute to the formation of highly GSH reactive metabolite 6-phenyl-3-hexen-2-one as its mercapturic acid adduct found in human urine [43]. In rat and human liver microssomes the electrophilic metabolites of kava, 11,12-dihydroxy-7,8-dihydrokavain-*o*-quinone and 11,12-dihydroxykavain-*o*-quinone, were described as glutathione conjugates [44]. These phase I quinone reactive metabolites, due to their capacity of forming covalent bonds with macromolecules containing nucleophiles groups or to their potential to induce redox cycling, were suggested to be implicated in liver injury. While it was not found mercapturic acids of these metabolites in the human urine, some authors evidenced the formation of glucuronic acid and sulfate conjugates of the catechols 11,12-dihydroxykavain and 11,12-dihydroxy-7,8-dihydrokavain [44]. It was hypothesized that these phase II metabolic routes may represent detoxification steps that preclude the oxidation of catechols to *o*-quinones [44].

### 5.2. Interactions and Metabolic Induction or Inhibition

Kava extract can significantly modulate drug-metabolizing enzymes, particularly the cytochrome P450 isozymes, a fact that has been suggested to predispose to drug-induced liver injury (DILI) [36,45]. Particularly kavalactones inhibition of CYP450 enzymes may predispose to relevant pharmacokinetic interactions [45].

At least seven genes of the cytochrome P450 isozymes were changed after kava extract exposure by gavage for 14 weeks [36]. Particularly, it increased the gene expression of *CYP1A1*, *CYP1A2*, *CYP2C2*, *CYP3A1*, and *CYP3A3*, while gene expression of *CYP2C23* and *CYP2C40* decreased, all in a dose-dependent manner. Clayton et al. [46] also reported a decreased expression of *CYP2D1* (human CYP2D6 homolog) in females and an increased expression of *CYP1A2*, *CYP2B1*, and *CYP13A1* of both sexes of Fischer 344 rats. Although these results might add further insights on the kava involvement in liver injury as hepatic hypertrophy was registered, the mechanisms remain largely unknown and several hypotheses have been proposed as described below.

The effects of the six major kavalactones on cDNA-derived CYP450 isoenzymes were evaluated by Zou et al. [47] who demonstrated that desmethoxyyangonin exhibited the most potent inhibition on CYP1A2. In the case of CYP2C19, the major inhibitors were dihydromethysticin, desmethoxyyangonin and methysticin, while for CYP3A4, methysticin and dihydromethysticin evidenced the best inhibitory effects. Noteworthy, kavain, the most potent anxiolytic kavalactone, as well as dihydrokavain and yangonin were largely ineffective in the highest tested concentrations.

Russmann et al. [48] also demonstrated that traditional kava drinking inhibits CYP1A2 expression, suggesting that metabolic interactions may occur with the concomitant administration of drugs that are CYP1A2 substrates such as theophylline, warfarin, fluvoxamine, and tizanidine. Moreover, since CYP1A2 is involved in the bioactivation of hepatic carcinogens such as aflatoxins, this fact points to a protective effect against environmental carcinogens especially in the warm and humid climate, where aflatoxin exposure is even more problematic. Indeed, in a study that will be further addressed in Section 8, Steiner [49] suggested an inverse correlation between cancer incidence and kava consumption for a number of Pacific Island Nations. It was also demonstrated that the kava extract induces CYP1A1 expression in a concentration-dependent manner, especially due to methysticin, but also 7,8-dihydromethysticin [50]. This induction suggests a possible role of kava or kavalactones in the bioactivation of polycyclic aromatic hydrocarbons (PAHs). Mathews et al. [35] observed that kava extract or a mixture of the 6 six major kavalactones present in the extract inhibited the expression of CYP2C9, CYP2C19, CYP2D6, CYP3A4 in human hepatic microsomes. Results also evidenced that kava extract and kavalactones possess a week induction on P-glycoprotein ATPase activity. In healthy volunteers, comparisons between the presupplementation and postsupplementation phenotypic ratio means revealed that kava causes a reduction in CYP2E1 expression of approximately 40% [51]. In addition to CYP isoenzymes, kava extract also demonstrated an inhibitory reversible effect on monoamine oxidase (MAO)-A and MAO-B activities of mouse brain, leading to research interest in the potential anti-parkinsonian and antidepressant activity of kava [52,53]. In vitro kava and its kavalactones were found to produce reversible inhibition of carboxylesterase 1 (CES1) either by a competitive and non-competitive subtype [54]. Hypothetical clinical implications are expected namely in the metabolism of several drugs administered concomitantly such as methylphenidate, clopidogrel and oseltamivir.

Since kava could be administered in conjunction with alcoholic drinks, this mixture can have important clinical, forensic, and social consequences in comparison to traditional kava consumption. Foo and Lemon [55] evaluated in humans the effect of ethanol or kava separately and in combination on subjective measures of impairment and intoxication and on cognitive performance. Kava alone produced no significant effects on perceived or measured competence, while ethanol caused motor and cognitive impairments in the five subjective measures evaluated. When ethanol was combined with kava, even larger negative changes were registered, which is also in accordance to mice reported studies [56]. Nevertheless, in a randomized trial involving 40 volunteers, no negative performance effects of combining kava with ethanol compared to kava alone were observed on the seven safety-related performance parameters analyzed [57]. Finally, in a recent study in the rat model, with the objective to elucidate the mechanisms of toxicity alone and in combination with ethanol, alterations of hepatic color, size, consistency, weight and necrosis with Kupffer cells hyperplasia of periacinar zone were all aggravated by ethanol [58].

## 6. Kava-Induced Toxicity and Safety Issues

### 6.1. Hepatotoxicity

As stated before, many people who use herbal supplements somehow perceive them as devoid of adverse side effects due to their natural origin. In this sense, these supplements might be used even when not needed, at higher doses than recommended and for long periods of time. It is well known that different types of toxic effects can be induced by herbal medicines, especially liver injury.

From the different types of toxic effects attributed to kava, hepatotoxicity is indeed considered the most dangerous and debated one. Cases of hepatotoxicity associated with kava intake started in the 1990s and by 2001—as mentioned above, BfArM announced the decision to ban kava, making it official in 2002, supported by 41 reports of liver injury in Germany and 73 cases worldwide [3,4]. This decision was altered in 2014 [1]. Since then and after some standardization rules were applied, the number of hepatotoxicity reports has considerably decreased. The guideline proposed by the WHO [59] had a significant impact in clinical trials, although not all cases reported during the 2000s were clearly associated with kava. In fact, as stated by Fu et al. [37], there is some degree of uncertainty in terms of the doses of kava used and the period of use.

Several putative causes for liver toxicity have been pointed out, ranging from the potential low-quality cultivars and inadequate extraction solvents to possible interactions with other drugs. Kava overdose can be associated with necrotic hepatitis and cholestatic hepatitis [60]. In such cases, nearly 80% of the individuals consumed 2-fold the maximum daily recommended dose and consumed kava for a prolonged time (more than 3 months up to two years), as mentioned in the WHO report [59]. This report was essential to help clarifying some pertinent issues, evaluating more than 90 hepatotoxicity episodes, and determining in which cases kava was considered responsible for the liver injury present. All the cases evaluated in this report were categorized as “probable” and “possible” and associated with hepatic pathologies. A case was considered “probable” when “good” adequate information was available (i.e., duration to the event, recovery on withdrawal of the product, absence of other potential causes of hepatotoxicity). Classification as “possible” reflected lack of information or the concomitant use of ethanol or drugs that might cause liver damage, albeit the impact of kava could not be excluded. Of all the cases evaluated, only 8 were considered “probable”, of which half used ethanol as a solvent extract, and 54 were considered as “possible”. Disregarding the 37 cases in which the identification of the solvent used was undetermined, it was reasonable to establish, for the cases where the extract used was ethanol or acetone, a “probable” or “possible” hepatic event. The most common hepatic events were hepatitis and hepatic failure. Of all the 93 cases, only 6 (6.25%) where associated with kava extracted with water. Due to an extensive lack of information, 28 cases were considered not assessable [59].

Among the different chemicals/classes of chemicals claimed to be responsible for kava hepatotoxicity, pipermethystine, flavokavain B and mold hepatotoxins have been pointed out in the literature. Pipermethystine is a piperidine alkaloid, present mainly in aerial parts of kava, such as leaves and peelings of the lower stem [61]. In vitro studies reported the loss of 90% cell viability by exposing the HepG2 cells to 100 µM of pipermethystine and 65% of cell death using 50 µM [62]. A recent in silico study showed that alkaloids and chalcones are similar in terms of water solubility and lipophilicity to kavalactones, which are lipophilic compounds, and therefore can be present in aqueous extracts, in minimum concentrations for the development of toxicity [63]. Despite its toxicity potential, pipermethystine may not be the responsible for the hepatotoxicity reports in Germany in the 2000s, as stated by Lechtenberg et al. [64], since the analysis of kava products with ethanolic extracts in the German market did not detect this alkaloid above the limit of quantification (45 ppm).

Another possible compound responsible for hepatotoxicity is flavokavain B. In vitro studies demonstrated cytotoxic activity in HepG2 cells and human liver cells L-02, leading to cell death, and demonstrated in in vivo studies that flavokavain B is responsible for the potentiation of kava hepatotoxicity upon herb–drug interaction in C57BL/6 mice. [65,66]. Nevertheless, it is still unclear if the results regarding cytotoxicity in vitro can be translated to human patients [61]. Other responsible compounds suggested in the literature include aflatoxins and ochratoxin A, which are not constituents of the plant, but rather possible contaminants. This is also a controversial possibility. While it is claimed that mold can develop and degrade kava in the containers and bags used for its transport to Europe, there is no strong evidence supporting this assumption. Moreover, the aflatoxins detected in kava are at concentrations considered too low to cause liver damage [61,67]. Despite all these possibilities, it should also be considered the combined toxic effect of different kava constituents. In fact, instead of being caused by only one single component, hepatotoxicity can be the result of an association of different compounds, a hypothesis that needs to be further addressed.

In addition to the abovementioned factors possibly responsible for kava-induced hepatotoxicity, i.e., extraction solvents used, misuse of other kava parts and possible toxic constituents, there are other aspects that should be considered for the liver injury observed, including the depletion of the reduced glutathione (GSH). In fact, the decrease in GSH contributes to the sensitization of liver to hepatotoxic agents [65]. It has been reported that flavokavain B depleted GSH and the presence of exogenous GSH protected liver cells from the induced cell death caused by this kava constituent. In a different model, amoeba cells treated only with kavalactones demonstrated high toxicity, leading to total cell death. Importantly, in the presence of GSH, only 40% of cells did not survive [68]. Therefore, it is reasonable to assume that GSH provides protection against kavalactones, via Michael reaction [45,68], and its presence is key to reduce hepatotoxicity.

The differences in the metabolism between Pacific islanders and Caucasians may also play a pivotal role, since it was suggested a possible correlation between kava-induced hepatotoxicity and CYP2D6 deficiency [69]. As approximately 12–21% of Caucasians are poor CYP2D6 metabolizers, compared to only 1% of Pacific islanders, this may explain the higher incidence of hepatotoxicity in Western countries [45]. Therefore, kava-related hepatotoxicity may be caused by different factors that combined lead to liver injury, factors that may or not be inherent to kava.

### 6.2. Other Adverse Effects on Consumers

While hepatotoxicity can be considered the main health concern, there are other side effects associated with kava ingestion that should be mentioned, with most of them being reversible. An example is kava dermopathy, also known as kavaism. It is a systemic dermatitis and erythema that can be observed first in the head, spreading down to the body (arms, body, neck, and breast), and resulting in a dry, scaly, and yellow discoloration [7,60,70]. It is more common in heavy kava consumers, being reversible upon the cessation of the intake of kava. In Fiji, where kava is consumed traditionally in higher amounts, this effect is common and seen as a privilege [7,18].

Other common side effects are gastrointestinal discomfort, nausea, and headaches. These effects have been described as reversible upon interruption of the kava treatment [30,71,72]. There are a few reports available addressing rare effects induced by kava that might be dependent on genetic predispositions or on the psychological state of the consumer [3]. Meseguer et al. [73] reported a case of kava-induced Parkinsonism in a 45-year-old woman that took 65 mg/day of kava for 10 days and rapidly triggered progressive parkinsonism for 3 months. Usually, movement associated pathologies appear upon the first use of the extract, which was not the case. This adverse effect was attributed either by the prolonged intake or by some type of genetic predisposition, since this patient had a family history of essential tremor [73]. The influence of the psychological state was addressed by Vignier et al. [74] who reported a possible relationship between suicidal behavior and kava drinking in teenagers from New Caledonia. The report showed a suicidal behavior in Kanak teenagers that ingested high quantities of kava when compared with non-Kanak, which might indicate a depressant effect induced by kava along with its anxiolytic properties. It is important to mention that there are both pharmacokinetic and pharmacodynamic drug interaction concerns associated with kava, especially with drugs that metabolized by CYP1A2, CYP2C19, and CYP3A, eliminated by P-glycoprotein or that have overlapping sedative or hepatotoxic effects [18].

Since kava might be an alternative treatment option for different diseases, it has been evaluated its potential impairment in terms of driving capacity. Some studies indicate that it may improve memory and attention, as mentioned in a clinical trial assessing driving ability [71]. Another study determined that medicinal quantities of kava did not affect the driving ability and cognitive performance [55], although Wainiqolo et al. [75] described an impact in driving from kava consumption in Fiji, upon the use of high amounts of kava, i.e., 50-fold higher than the used in herbal and therapeutic preparations.

### 6.3. Genotoxicity and Carcinogenicity Potential

Studies addressing the genotoxicity and carcinogenicity of kava extracts have been reported in the literature, with most of them being recently compiled and discussed by the IARC [6]. Kava extracts displayed negative results in several reverse mutation assays in bacterial systems (*Salmonella typhimurium* TA97, TA98, TA100 and TA 1535 and *Escherichia coli* strain WP2 uvrA pKM101), either in the presence or absence of external metabolic activation (rat S9-mix), as reported by the NTP [5]. Kavalactones displayed negative results in most of these assays [6]. Whittaker et al. [76] also reported negative results in the L5117Y mouse lymphoma assay performed with human liver S9 for both kava products and kavalactones. The IARC monograph also included the study from Jhoo et al. [77], in which two positive results for kava extracts were found using the umu mutation assay. In this study, however, six negative results were also observed and the IARC working group mentioned the absence of statistical analysis of the data [6]. NTP also reported negative results in the in vivo micronucleus assay performed with B6C3F mice (male and female) treated with kava extracts for 3 months [5]. Recently, the mutagenic and carcinogenic potential of kavain and its combined effect with doxorubicin (Dox) was assessed in the *Drosophila melanogaster* model [78]. In the crosses with basal CYP450 metabolism, kavain was not mutagenic in the Somatic Mutation and Recombination Test (SMART). However, this compound exhibited an indirect mutagenic activity at the higher concentrations tested in the crosses that display high CYP450 activity. At this concentration level, kavain also increased the mutagenic activity of Dox, while at lower concentrations displayed, in contrast, protective effects upon combination with this chemotherapeutic drug. The authors also studied the carcinogenic and anticarcinogenic potential of kavain alone using the Epithelial Tumor Test and did not find clear positive responses.

In what concerns the carcinogenicity potential of kava extracts, positive results for kava extracts delivered by oral gavage were reported in long-term studies carried out by NTP [5]. Particularly, in the 2-year studies these extracts revealed in male B6C3F1 mice a clear evidence of carcinogenicity activity, with increases in the incidence of hepatoblastoma. In female mice from this strain some evidence of carcinogenicity was found in terms of hepatocellular adenoma and carcinoma (combined). In male F344/N rats marginal increases in terms of testicular interstitial (Leydig) cell adenoma were found albeit the report considered this as equivocal evidence of carcinogenic activity. No evidence was reported in female rats treated with this extract. In sum, according to the IARC [6], there is inadequate evidence in humans for the carcinogenicity of kava extract but sufficient evidence in experimental animals which resulted in the overall classification in Group 2B—possibly carcinogenic to humans. Moreover, the carcinogenicity of kava reported in the mice model was considered most likely to occur through a non-genotoxic mechanism as noted by the IARC [6].

## 7. Clinical Potential Uses of Kava in Non-Cancer Diseases

As previously mentioned, several compounds of kava, such as kavain, dihydrokavain, methysticin, desmethoxyyangonin and yangonin are able to cross the blood–brain barrier in in vivo studies [39]. Kava generally generates a soothing and relaxing sensation [11,79,80]. In view of this, it has been primarily employed in clinical trials to treat anxiety. In fact, the medicinal uses of kava supported by clinical data are the short-term treatment of mild types of anxiety and insomnia. Moreover, as reviewed by the IARC [6], other clinical uses in the medical literature include the reduction in body weight and the management of fungal infections. Albeit not supported by scientific evidence, in traditional medicine, kava has been used in several other diseases, including asthma, different types of infections, headache, and menstrual disorders [6].

The clinical and experimental data on kava psychopharmacology were also recently reviewed by Volgin et al. [81], highlighting the potent effects of kavalactones as the primary psychoactive chemicals in kava. Additionally, a systematic review on randomized clinical trials was published regarding the effectiveness and safety of kava in anxiety [82]. The authors claimed the use of kava as a short-term solution for anxiety since it was more effective than placebo in three of the seven trials and the adverse effects were similar to the placebo. However, these authors did not suggest such beneficial role for its long-term use.

In some clinical trials performed, the participants in the kava group showed improvement in terms of stress and anxiety, when compared to the placebo group, with no serious side effects reported [29,83]. Nevertheless, a clinical trial showed no changes in the kava group, regarding the reduction in anxiety, whereas the oxazepam group displayed a significant reduction [84]. In general, these more recent clinical trials herein mentioned can be regarded as valuable to determine whether kava is efficient and safe to be considered a treatment option, since most of them follow the WHO guidelines. These guidelines encompass the use of an aqueous extract kava (only the root or rhizome) in participants without previous liver disease or history, ethanol abuse and exclude participants medicated with antipsychotics, anxiolytics or antithrombotic drugs [59]. Volz and Keiser [85] have previously reported a clinical trial with kava focusing on its anxiolytic effects. This study had a long duration (six months) and large sample (101 patients), thus being considered relevant in this context. During this randomized controlled trial (RCT) trial, the patients received the WS 1490 kava extract, which contained 70 mg of kavalactones, with this extract being administered orally three times a day. In this study, anxiety was assessed by Hamilton Anxiety Rating scale (HAMA) score and decreased significantly in the kava group, compared with the placebo group. Despite the large amount of kava administered, no serious side effects or liver damage were observed, with stomachache being the most common side effect encountered.

Kava has been compared to standard anxiolytic drugs, such as oxazepam, to determine if it may have any withdrawal or addiction issues [71,84]. According to these studies, kava displays no addiction or withdrawal events at the dosage and time used, unlike classical anxiolytics. In these clinical trials, nausea, stomach upset, headaches and dermatitis were the most common adverse effects, with most of them being reversible by cession kava intake or lasting just a few days. Liver hepatotoxicity was not mentioned. These data suggest a good safety profile for short-term (≤6 weeks) and dosage (≤250 mg kavalactones/day). These aspects were tightly controlled in the abovementioned studies, limiting their translation to other scenarios (e.g., recreational potential) where the interaction with other compounds or influence of concomitant diseases may lead to different conclusions [18,29].

As aforesaid, although the focus of kava is to treat anxiety disorders, it has been also used to mitigate disorders such as menopause symptoms and associative depression [86,87] and insomnia [88,89]. There is also evidence that kava may improve women’s libido, due to kava anxiolytic effect, rendering women more susceptible to sexual intercourse as described in an Australian clinical trial [72]. In contrast, opposite effects were observed in men from Tonga island [12]. Additional information on this topic regarding studies with populations from other countries is needed. In addition, traditional reports mentioned that men that consume kava are more likely to miss work [12].

As previously mentioned, kava has shown an effect in the CNS, mainly due to kavain, dihydrokavain, methysticin, desmethoxyyangonin and yangonin. These kavalactones were able to cross the blood–brain barrier in in vivo studies [39]. Despite the kava implication in psychotic syndromes and possible seizures [90], some studies have been conducted in animals to assess its possible anticonvulsant effect. Volgin et al. [81] noted that, in the beginning of the 1960s, in a study performed by Meyer, kavalactones were administered orally or intraperitoneally and displayed sedative and anticonvulsant effects in several animals (mice, rats, rabbits, and cats). In this context, Tawfiq et al. [91] investigated the effect of kava alone or in combination with an anticonvulsant drug, diazepam, in female Wistar rats. Kava enhanced the anticonvulsant effect of diazepam at 100 and 200 mg/kg and improved the reduction in locomotor activity and liver function tests induced by diazepam. Another in vivo study evaluated the anticonvulsant effects of kavas’ stem peelings in the zebrafish model. An aqueous extract of kava without peel after 45 min of pre-treatment showed an anticonvulsant potential at a dose of 50 mg/L [92]. Another clinical trial develops a kava-based intervention to enable tobacco cessation and reduce lung cancer risk in active smokers who have no intention to quit smoking (www.clinicaltrials.gov, accessed on 22 February 2022).

## 8. Kava in Cancer Prevention and Treatment

### 8.1. Potential Roles of Kava in Cancer

The carcinogenic potential of kava and its constituents was addressed in previous Section 6. Herein, we review and discuss a number of publications that, in general, point out to important features towards an opposite effect, that is a beneficial role of kava and its constituents in cancer prevention and treatment. According to the IARC report [6], albeit the sufficient evidence for carcinogenicity in rodent models there is an inadequate evidence for this outcome in humans. The data available supported the overall classification in category 2B, which means that kava studies did not provide the requisites necessary for the classification in categories of higher concern (i.e., categories 1 and 2A). Moreover, it should be stressed that carcinogenic effects observed in experimental animals were most likely attributed to non-genotoxic mechanisms. While the evidence of kava as an animal carcinogen should always be considered with caution, this do not preclude the interest of the scientific community to study kava and its constituents as bioactive agents in cancer.

In the late 1990s, three studies first reported low cancer rates, especially lung cancer, in the Pacific islands inhabitants, linking this interesting data with chemopreventive elements in diet [93,94,95]. Based on these studies, Steiner established an inverse relationship between cancer rates and kava consumption, observing lower incidence rates of cancer in men compared with women [49]. These findings were somehow in contrast with the expected rates in view of the higher consumption of kava by men. This article was mentioned in the IARC report, although it was considered uninformative in view of its ecological design, being the inadequate statistical assessment highlighted by the working group [6]. Nevertheless, since the Steiner study [49], several experimental studies have been conducted to evaluate the effectiveness of kava in diverse cancers, including the most common ones (e.g., prostate, lung, colorectal and breast cancers), either using kava extracts or specific components of it and, in many cases, the effect of kava could be considered meaningful. Recently, Celentano et al. [15] conducted a systematic review on the possible anticancer effects of kava components. These authors concluded that some specific kava constituents regularly demonstrate anticancer properties, with flavokavain B and A being the most studied. These components can act by increasing or decreasing the expression of pro-apoptotic and antiapoptotic proteins, respectively.

In this section, the main compounds suggested to be implicated in these beneficial effects in cancer are addressed in more detail. The usefulness of kava in the context of cancer can be regarded by two complementary perspectives: chemoprevention or, alternatively, as a therapeutic agent. It is well known that chemotherapeutic drugs can also arise from natural sources, nearly representing half of the anticancer agents according to Newman and Cragg [96]. Moreover, the main purpose of chemoprevention is to reduce or delay the initiation of carcinogenesis [15]. For an agent to be considered chemopreventive, some authors advocate that it needs to fulfill certain criteria including low toxicity, with few or no adverse side effects, easy to ingest and/or administer and a low cost [97]. Kava and its components have demonstrated antiproliferative, antimetastatic and apoptosis-inducing mechanisms, displaying properties of a putative chemopreventive or chemotherapeutic drug.

### 8.2. Anticancer Properties of Kava Extracts

Different types of kava extracts and the impact of extraction solvents in their chemical composition and cytotoxic effects have been evaluated either in in vitro or in vivo models. It has been shown that it is important to examine the activity of the plants in the traditional form, for example using water instead of ethanol or acetone to extract components [98]. Einbond et al. [99] showed that adding sea hibiscus bark to kava extracts can enhance the inhibitory growth effect of kava in HT29 colon cancer cells. This combination is used in Pohnpei and Fiji preparations to squeeze the extract [99].

Despite some studies showing anticancer or chemopreventive proprieties of kava extracts in the context of lung cancer [100,101], there is a study that failed to show this beneficial effect [98]. Accordingly, aqueous extracts from 25 commercial kava samples in concentrations up to 500 mg/mL showed no cytotoxic impact in human lung adenocarcinoma A549 cells, indicating low or even no anticancer potential when water was used as the extraction solvent in this cancer cell line.

Kava extracts tend to be more often studied in vivo than in vitro to assess the synergistic effect of different kavalactones with flavokavains in cancer endpoints, and to evaluate whether they can induce liver toxicity or injury. It is frequent to separate kava extracts into fractions to determine which one is more effective. By separating it into polar and non-polar fractions, Triolet et al. were able to successfully reduce morphological markers, aberrant crypt, and foci in carcinogen-treated rats for twelve days, specifically with the non-polar fraction [102]. Leitzman et al. separated a kava extract in three fractions (A, B and C) using HPLC to determine which active fraction, and consequently which components, could be responsible for kava´s chemopreventive efficacy in A/J mice in a 4-(methylnitrosamino)-1-(3-pyridyl)-1-butanone (NNK)-induced lung tumorigenesis model [100]. Fraction B was the only one capable of reducing adenoma incidence by 93% and adenoma multiplicity to baseline level. This fraction contained six kavalactones and one flavanone, indicating that kavalactones might be the main responsible for the kava´s chemopreventive properties. In both studies, liver weight was measured, and no significant changes were detected.

In addition, a long-term study in which kava extracts were administered for 22 weeks to A/J mice (oral administration) did not find significant effects on liver integrity enzymes or liver weight [103]. Tobacco smoke contains the carcinogen NNK. This compound, when reduced to 4-(methylnitrosamino)-1-(3-pyridyl)-1-butanol (NNAL), can bioactivate reactive species that damage DNA [104]. Some DNA adducts are not stable and can be extracted through urine. 3-Methyladenine (3-mA), a DNA adduct, appears to be tobacco-dependent and a biomarker of NNK activation [105]. Since dihydromethysticin has already proven to be highly effective in A/J mice [101], it is hypothesized that dihydromethysticin might reduce NNK/NNAL-induced DNA damage and potentially enhance NNAL urinary detoxification.

A recent pilot clinical trial [105] evaluated the impact of kava in human lung cancer, more specifically, the impact of kava extracts on biomarkers of tobacco usage and nitrosamine-based carcinogenesis in active smokers. Upon a 7-day course of kava, it was shown that the urinary excretion of NNAL increased, and 3-mA reduced, suggesting the ability to reduce the formation of NNK. It was also possible to detect lower levels of plasma cortisol and urinary total cortisol equivalents, both indicative of reduced tobacco use, possibly due to kava’s anti-stress activity [105].

### 8.3. Anticancer Properties of Flavokavains

Among the different types of chemicals present in kava, flavokavains have been evaluated both in in vitro and in in vivo models for their possible anticancer proprieties. Flavokavain A, B and C are at a minor percentage in kava, constituting up to 0.46%, 0.015% and 0.012%, respectively [106]. Although representing only less than 1% of the components of the plant, these chemicals have been studied by different research groups. Flavokavains are chalcones and, as such, can be easily synthesized [107]. Considering the 16 studies present in Table 1, eight are related to flavokavain B, six to flavokavain A and only two mentioned flavokavain C. In the studies with flavokavain A, the most common effect was the induction of apoptosis and antiproliferative effects by G2/M cell cycle arrest [108,109]. One study showed cell cycle arrest in the G2/M and G1 phases in MDA-MB-231 and MCF-7 cells, respectively, suggesting a p53 dependency [110]. However, another study concluded that the activation of Bax by flavokavain A, in an invasive bladder cancer cell line T24, might be p53 independent [106]. In view of this, future studies are necessary to determine the effects of flavokavain A on the p53 tumor suppressor-dependent pathways. It was also reported the effect of flavokavain A in the extrinsic and intrinsic apoptosis pathways, since it can upregulate Bim, p23, DR5 and Bax and downregulate Ki67, survivin and XIAP in a dose-dependent manner [108,110,111].

The beneficial effects of flavokavain B are still controversial. In fact, although it has shown promising results, it has also been pointed out as responsible for some adverse effects, especially hepatotoxicity. Regarding the main in vitro effects of flavokavain B, its ability to induce cell cycle arrest in G2/M and apoptosis is generally reported [115,119,120]. The IC_50_ values varied between 12.3 and 33.8 μM, for the MDA-MB-231 and MCF-7 cell lines, respectively [114].

Although the mechanisms are yet to be fully understood, most of the proposed mechanisms involve extrinsic and intrinsic apoptotic pathways, where DR5, PUMA and Bim are upregulated and the expression of antiapoptotic proteins, such as survivin and Bcl-2 is diminished [119,120]. Flavokavain B can also increase the production of ROS and decrease the expression of PI3K and Akt, leading to the blockage of the PI3K/Akt pathway [118]. Two in vitro studies on breast cancer cell lines, using 4T1, MCF-7 and MDA-MB-231 cells, showed flavokavain B ability to influence inflammatory and metastasis processes [113,114]. This compound also inhibited the migration and invasion of different cancer cells [113,114,118], displaying possible antimetastatic ability [114], and these effects are in general concentration dependent.

The research on the impact of flavokavain C on cancer cells is still recent, being the least studied flavokavain. Cell cycle arrest was observed in the S phase in HCT 116 cells and in the G1 and G2/M phases in HT-29 cells [121,122]. Even though it uses extrinsic and intrinsic pathways, it upregulates p21 and p27, and downregulates XIAP, c-IAP1 and c-IAP2 [122]. In addition to the anticancer potential of these flavokavains per se, there are also studies where their combination with anticancer drugs was addressed. In such studies, flavokavains can increase drug activity by being able to pass the senescent state and achieve apoptosis [117], or increase their anticancer potential [108,116].

### 8.4. Anticancer Properties of Kavalactones

There are only a few reports on the effects of kavalactones alone in cancer. From these studies, it is possible to conclude that dihydromethysticin can be considered the most effective and promising chemopreventive kavalactone (Table 2), as well as the most studied one. Nevertheless, yangonin has also shown some promising results. As such, it was found to induce autophagy via inhibition of the mTor pathway, where beclin, ATG5 and LKB1 were upregulated and Akt, PRAS40 and 4E-BP1 were downregulated by decreasing its phosphorylation. This kavalactone was more effective in cells with high levels of tSC1/2 expression, therefore being dependent on the presence of TSC1, and proved to have a synergic effect when combined with flavokavain A or docetaxel [123].

The impact of dihydromethysticin on the formation of NNK-induced lung adenomas and adenocarcinomas in A/J mice has been reported. This animal model has predisposed pulmonary adenoma susceptibility gene (Pas1) and develop lung adenomas with high incidence [126]. Dihydromethysticin was extremely effective, leading to a temporally 100% inhibition of adenomas, when administered 3–8 h pre-NNK injection and a 52% inhibition, when administered 40 h earlier [101]. The racemic synthetic mixture of dihydromethysticin was as equally chemopreventive as natural dihydromethysticin [9]. A study regarding the structure evaluation of kavalactones, specially dihydromethysticin and dihydrokavain, determined that the methylenedioxy functional group of dihydromethysticin was determinant to its chemopreventive activity and the lactone functional group can be modified, while dihydrokavain is completely ineffective [127]. Most studies found no anticancer activity in dihydrokavain [9,127].

Despite being studied more in the scope of lung cancer, dihydromethysticin has also shown inhibitory effects in colorectal cancer, mainly in terms of proliferation, migration, and invasion. It was also shown to induce apoptosis and cell cycle arrest in the G0/G1 phase, via the NLRC3/PI3K pathway, where it activates nucleotide oligomerization domain-like receptor subfamily C3 (NLRC3) and inhibits PI3K and cyclin D1-CDK4, leading to G0/G1 arrest [124].

## 9. Final Considerations

**Figure 2:**
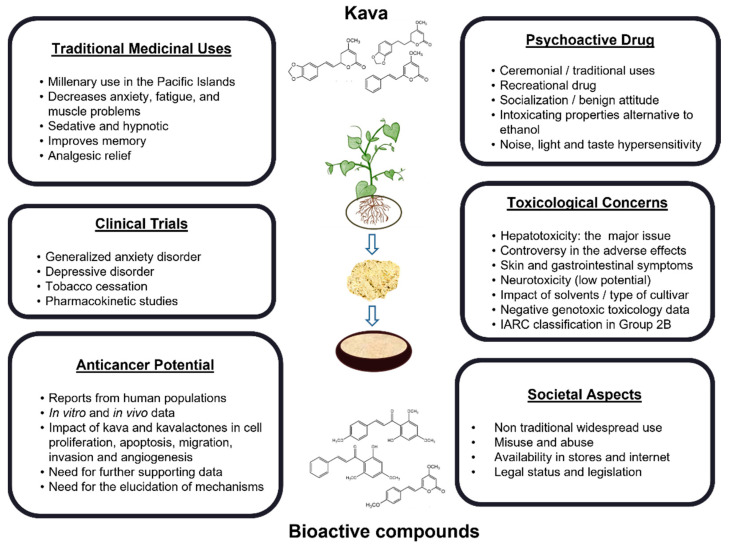
Different contexts in which kava can be studied.

Kava is undoubtedly a topic of great scientific interest and relevance that can be analyzed under different perspectives and contexts, as depicted in Figure 2. Herein, the complexity of kava is highlighted, showing the interest in kava in terms of clinical potential, toxicological concerns and as an emerging recreational drug with a millenary use. Kava gives a sensation of calm and relaxation to the consumers. These characteristics triggered the interest of Western countries and clinical trials have been conducted. However, due to its trivialization, safety challenges concerning the use of kava as an alternative medicine have also been emphasized. Despite the importance of the WHO guidelines to tackle this problem, there is still the need for a more rigorous standardization of the process of extraction and commercialization. These guidelines had a great impact on clinical trials and respective results, but the outputs obtained in controlled conditions are unfortunately not present in the regular consumption of kava. In this context, further information is needed for individuals that are exposed to large amounts of kava for longer periods of more than 4-6 weeks. The toxicity issues should be properly addressed towards the safer consumption of kava. There is also a clear need to identify the putative chemicals that may be involved in the toxic effects of kava. In this sense, some compounds have been mentioned in the literature. As such, flavokavain B might be responsible for herb–drug interactions in vivo [66], although, to the best of our knowledge, no human studies have been reported. On the other hand, pipermethystine, a water-insoluble substance mainly present in kava leaves has been pointed out as another possible source of human kava hepatotoxicity. However, there is still scarce in vitro evidence that sustains this possibility [61]. Moreover, the impact of mold hepatotoxins (e.g., aflatoxins) is still a controversial possibility [61,67]. Although the WHO guidelines clearly aid at mitigating these toxicological aspects, it is still important to assess this topic in all the studies or clinical trials conducted, and it is also necessary to provide standardized rules for the exportation process.

Overall, adverse toxic effects, particularly kava-induced hepatotoxicity, have been a concern regarding the use of kava as an anxiolytic or as a chemotherapeutic/chemopreventive agent against cancer. The highly complex roles of kava in the cancer field should be mentioned. Albeit not mutagenic, kava extracts are considered possibly carcinogenic to humans due to sufficient evidence obtained in experimental animals [6]. Nonetheless, human carcinogenicity data are inadequate and there is an obvious need for well-conducted cancer epidemiological studies with kava. On the other hand, there is growing evidence that specific kava components have interesting anticancer properties. In several studies, the use of kava extracts, or their isolated components, leads to the inhibition of cell proliferation, cell cycle arrest and induction of apoptosis, showing promising results against cancer. Finally, although the studies presented here pointed out kava as a promising chemotherapeutic or chemopreventive agent, it is still largely unknown how kava and its constituents interact with cancer cells and the molecular mechanisms involved, being this an emerging research topic.

## Figures and Tables

**Table 1:** Overview of recent studies with flavokavains using in vitro and in vivo cancer models.

Flavokavain	Cancer	Study Type	Possible Mechanisms Involved	Key Findings	References
Flavokavain A	Bladder cancer	In vivo	Upregulation of p27 and DR5 and downregulation of Ki67, survivin and XIAP	Inhibition of occurrence of high-grade papillary UCC by 42.1% and promotion of apoptosis in UPII-SV40T transgenic mice	[111]
	Bladder cancer	In vitro /In vivo	Bax protein-dependent and mitochondria-dependent apoptotic pathways	Inhibition of growth tumor cells by apoptosis (57% decrease) in xenograft mouse model	[106]
	Breast cancer	In vitro /Ex vivo	Intrinsic mitochondrial pathway with potential dependency on the p53 status	Cell cycle arrest G2/M in MDA-MB-231 and G1 in MCF-7. Induction of apoptosis in both cell lines	[110]
	Breast cancer	In vitro	Inhibition of Cdc2 and Cdc25C phosphorylation and upregulation of Bim and BAX	Cell cycle arrest G2/M. Flavokavain A in the presence of herceptin enhanced treatment. Induction of apoptosis in SKBR3.	[108]
	Lung cancer	In vitro	Downregulation of P-gp by inhibition of PI3K/Akt pathway	Inhibition of cell proliferation and induction of apoptosis of PTX-resistant A549/T cells in a concentration-dependent manner	[112]
	Prostate cancer	In vitro	Glutamine metabolism pathway upregulated, reducing the levels of glutamine, glutamic acid, and proline in PC3 cells	Reduced glutamine decreased GSH levels, which increased ROS levels and consequently cell apoptosis. Cell cycle arrest in G2/M	[109]
Flavokavain B	Breast cancer	In vitro	-	Inhibition of proliferation, migration, and invasiveness in 4T1 cells. Reduced weight and size tumors after 28-days of treatment in cell-challenge mice	[113]
	Breast cancer	In vitro /Ex vivo	Tyrosine kinase pathways	Induction of apoptosis and cell cycle arrest in G2/M in MDA-MB 231 and MCF-7 cells. Inhibition of migration and invasion in MDA-MB 231 cells and angiogenesis in HUVEC cells and in the rat aortic ring assay	[114]
	Colon cancer	In vitro	Cyclization of flavokavain B to 5,7-dimethoxyflavone	Inhibition of cell proliferation and cell cycle arrest in G2/M in LoVo and LoVo/Dx cell lines	[115]
	Gastric cancer	In vitro /In vivo	Extrinsic and intrinsic apoptotic pathways	Flavokavain B in the presence of doxorubicin suppresses cell growth and induces apoptosis and autophagy in BALB/c mice and in AGS cells	[116]
	Glioblastoma multiforme	In vitro /In vivo	Induction of autophagy	Inhibition of cell growth through autophagy in U251, U87 and T98 cell lines and combined with autophagy inhibitors led to apoptosis in mice	[117]
	Lung cancer	In vitro	Intrinsic apoptosis pathway and blockage of PI3K/Akt signaling pathway	Flavokavain B-induced apoptosis, ROS production and inhibits migration and invasion in A549 cell line	[118]
	Synovial Sarcoma	In vitro	Extrinsic and intrinsic apoptotic pathways	Inhibition of cell growth in SYO-I and HS-SY-II cell lines in a concentration-dependent manner	[119]
	Uterine Leiomyosarcoma	In vitro	Upregulation of DR5, Puma and Bin and downregulation of survivin	Cell cycle arrest in the G2/M and induction of apoptosis in SK_LMS-1 and ECC-1 cell lines	[120]
Flavokavain C	Colorectal cancer	In vitro	Induction of intrinsic and extrinsic apoptosis pathways by an inactivation of Akt pathway and modulation of MAPK pathway	High cytotoxicity in HCT 116 cells in a time- and concentration-dependent manner. Disruption of the mitochondrial membrane potential and cell cycle arrest in the S phase	[121]
	Colorectal cancer	In vitro	Inactivation of inhibitor of apoptotic proteins and endoplasmic reticulum stress pathways	Decreased cell viability and SOD activity and increased of ROS in HT-29 cells	[122]

XIAP, X-linked inhibitor of apoptosis protein; Cdc2, Cell-Division Cycle 2, Cdc25C, Cell Division Cycle 25C; Bax, Bcl-2-associated X protein; PI3K, Phosphoinositide 3-kinase; PTX, paclitaxel; ROS, reactive oxygen species; DR5, Death receptor 5; SOD, superoxide dismutase.

**Table 2:** Overview of recent studies with kavalactones using in vitro and in vivo cancer models.

Cancer	Study Type	Compound	Possible Mechanisms Involved	Key Findings	References
Bladder cancer	In vitro	Yangonin	Inhibition of mTOR pathway	Induction of autophagic cell death in UMUC-3 and T24 cells and growth inhibition in RT4, T24, UMUC3, HT1376 and HT1197 cell lines.	[123]
Colorectal cancer	In vitro/ In vivo	Dihydromethysticin	NLRC3/PI3K pathway	Inhibition of proliferation, migration, invasion and promotion of cell apoptosis and cell cycle arrest in HCT116, HT29 and LoVo. Inhibition of tumor growth in male BALB/C nude mice.	[124]
Lung cancer	In vivo	Dihydromethysticin	Inhibition of NNAL activation/increased NNAL detoxification	Reduction in adenocarcinoma multiplicity (97% decrease) and DNA adducts in A/J mice.	[9]
Lung cancer	In vivo	Dihydromethysticin	Inhibition of NNK-induced O6-mG	Temporally complete inhibition of lung adenoma in A/J mice. Pre-NNK administration of dihydromethysticin highly effective.	[101]
Osteosarcoma	In vitro	Dihydromethysticin	Decreased activity of PI3K/Akt pathway/disruption of MMP	Cell apoptosis and cell cycle arrest in G0/G1 in MG-63 cells. Inhibition of proliferation.	[125]

mTOR, mammalian target of rapamycin; NLRC3, nucleotide-oligomerization domain-like receptor subfamily C3; PI3K, Phosphoinositide 3-kinase; NNAL, 4-(methylnitrosamino)-1-(3-pyridyl)-1-butanol; NNK, 4-(methylnitrosamino)-1-(3-pyridyl)-1-butanone; MMP, Matrix metalloproteinase.
